# Intensity of and Adherence to Lipid‐Lowering Therapy as Predictors of Major Adverse Cardiovascular Outcomes in Patients With Coronary Heart Disease

**DOI:** 10.1161/JAHA.122.025813

**Published:** 2022-07-05

**Authors:** Faizan Mazhar, Paul Hjemdahl, Catherine M. Clase, Kristina Johnell, Tomas Jernberg, Arvid Sjölander, Juan Jesus Carrero

**Affiliations:** ^1^ Department of Medical Epidemiology and Biostatistics Karolinska Institutet Stockholm Sweden; ^2^ Department of Medicine Solna, Clinical Epidemiology Unit, Karolinska Institutet and Clinical Pharmacology Karolinska University Hospital Stockholm Sweden; ^3^ Department of Medicine and Health Research Methods, Evidence and Impact McMaster University Ontario; ^4^ Department of Clinical Sciences Danderyd University Hospital, Karolinska Institutet Stockholm Sweden; ^5^ Division of Nephrology Department of Clinical Sciences, Karolinska Institutet, Danderyd Hospital Stockholm Sweden

**Keywords:** adherence, intensity, lipid‐lowering treatment, MACE, Secondary Prevention

## Abstract

**Background:**

The effectiveness of lipid‐lowering therapy (LLT) is affected by both intensity and adherence. This study evaluated the associations of LLT intensity, adherence, and the combination of these 2 aspects of LLT management with the risk of major adverse cardiovascular events (MACE) in people with coronary heart disease.

**Methods and Results:**

This is an observational study of all adults who suffered a myocardial infarction or had coronary revascularization during 2012 to 2018 and initiated LLT in Stockholm, Sweden. Study exposures were LLT adherence (proportion of days covered), LLT intensity (expected reduction of low‐density lipoprotein cholesterol), and the combined measure of adherence and intensity. At each LLT fill, adherence and intensity during the previous 12 months were calculated. The primary outcomes were MACE (nonfatal myocardial infarction or stroke and death); secondary outcomes were low‐density lipoprotein cholesterol goal attainment and individual components of MACE. We studied 20 490 patients aged 68±11 years, 75% men, mean follow‐up 2.6±1.1 years. Every 10% increase in 1‐year adherence, intensity, or adherence‐adjusted intensity was associated with a lower risk of MACE (hazard ratio [HR], 0.94 [95% CI, 0.93–0.96]; HR, 0.92 [95% CI, 0.88–0.96]; and HR, 0.91 [95% CI, 0.89–0.94], respectively) and higher odds of attaining low‐density lipoprotein cholesterol goals (odds ratio [OR],1.12 [95% CI, 1.10–1.15]; OR, 1.42 [95% CI, 1.34–1.51], and OR, 1.16 [95% CI, 1.19–1.24], respectively). Among patients with good adherence (≥80%), the risk of MACE was similar with low‐moderate and high‐intensity LLT despite differences in the low‐density lipoprotein cholesterol goal attainment with the treatment intensities. Discontinuation ≥1 year increased the risk markedly (HR,1.66 [95% CI, 1.23–2.22]).

**Conclusions:**

In routine care, good adherence to LLT was associated with the greatest benefit for patients with coronary heart disease. Strategies that improve adherence and use of intensive therapies could substantially reduce cardiovascular risk.

**Nonstandard Abbreviations and Acronyms**
LLT: lipid‐lowering therapy
MACE: major adverse cardiovascular events
PDC: proportion of days covered

**Clinical Perspective**

**What Is New?**

- Both adherence and treatment intensity can alter the effectiveness of lipid‐lowering therapy (LLT) in routine clinical practice; suboptimal LLT management may affect cardiovascular disease recurrence and low‐density lipoprotein cholesterol (LDL‐C) goal attainment.
- This study evaluated a combined measure of adherence and treatment intensity using a roll‐out design, and we assessed adherence at each LLT fill. This minimizes the immortal‐time bias of evaluating treatment adherence within fixed periods.
- We controlled for LDL‐C levels and the frequency of LDL‐C testing, which are important time‐dependent confounders, because regular LDL‐C monitoring may indicate health‐seeker or compliant behaviors and motivate treatment decisions.

**What Are the Clinical Implications?**

- Optimal LLT use had a significant prognostic benefit, regardless of treatment intensity, in post–myocardial infarction or revascularized patients with coronary heart disease.
- Good adherence to LLT was more important than LLT intensity or achieved LDL‐C levels for the reduction of cardiovascular risk in patients with coronary heart disease.
- The highest cardiovascular risk was observed among those who discontinued LLT. Strategies that improve adherence and greater use of intensive therapies could substantially reduce cardiovascular risk in secondary prevention.

Patients with established coronary heart disease (CHD) require targeted risk management strategies, including effective lipid‐lowering therapy (LLT) because of their high rates of subsequent cardiovascular events and death (ie, secondary cardiovascular prevention).^1^ Clinical trials have demonstrated the benefit of treating with statins, particularly with high‐intensity statins^2^ and, when needed, with further intensification by nonstatin LLT.^3^, ^4^ Because each 1 mmol/L (38.6 mg/dL) reduction in low‐density lipoprotein cholesterol (LDL‐C) is associated with a 20% relative reduction of major cardiovascular events in clinical trials,^1^, ^5^ long‐term adherence to and persistence on effective LLT is strongly recommended by clinical guidelines.^6^

However, observational studies from routine care show that many patients do not receive high‐intensity LLT as first‐line therapy, fail to be switched to high‐intensity LLT, and have suboptimal adherence rates.^7^, ^8^, ^9^, ^10^ Suboptimal LLT management may affect cardiovascular disease (CVD) recurrence and LDL‐C goal attainment, but this is not well studied. Some studies have explored the separate or combined associations between LLT adherence, intensity, and poor clinical outcomes.^11^, ^12^ Limitations of previous studies include fragmentation of health care (ie, use of primary health care records only), heterogeneous indications for LLT use (eg, diabetes or different subtypes of CVDs), lack of information on LDL‐C levels; and evaluation of fixed periods of adherence (eg, adherence during the first year of therapy), which may induce immortal time bias, because patients need to be on treatment and alive to be evaluated.

## METHODS

The data analyzed in this study are available from the corresponding author upon reasonable request.

### Data Sources

We used data from the Stockholm Creatinine Measurements database, a health care use database of residents in Stockholm, Sweden.^13^ In brief, the Stockholm Creatinine Measurements captures the complete health care use (including primary health care) and a wide range of routine laboratory test results among individuals who had plasma/serum creatinine or albuminuria measured at least once in the Stockholm region during 2006 to 2019. It has previously been estimated that around 98% of patients with CVD in the region are covered by the Stockholm Creatinine Measurements.^13^ Using the unique personal identification number of each inhabitant in Sweden, these data were linked to various regional and national administrative databases providing information on health care use, including *International Classification of Diseases, Tenth Revision* (*ICD‐10*) codes for all hospital admissions, causes of death, and dispensed prescription drugs. All diagnoses were coded according to the *ICD‐10*. The regional ethical review board in Stockholm approved the study (EPN 2017/793–31) and waived the need for informed consent, because data made available to researchers were deidentified.

### Cohort Definition and Study Design

**Figure 1:**
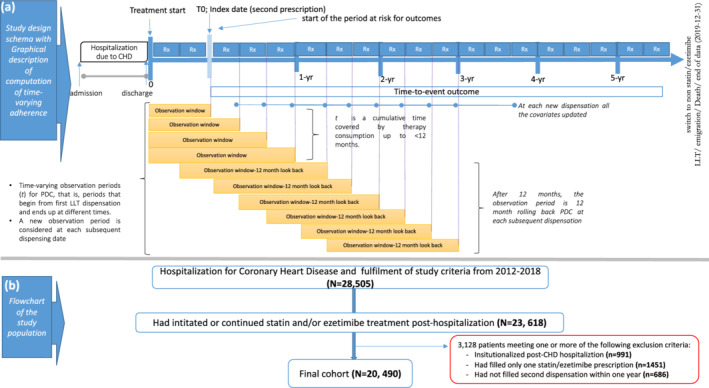
Study design schema (**A**) and flowchart of study inclusion according to Consolidated Standards of Reporting Trials (**B**). CHD indicates coronary heart disease; LLT, lipid‐lowering therapy; and PDC, proportion of days covered.

We selected all adult patients (aged ≥18 years) admitted for a first or recurrent CHD event, including hospitalization for acute myocardial infarction or coronary revascularization (coronary artery bypass graft surgery or percutaneous coronary intervention) (see Data S1) and who initiated statin and/or ezetimibe treatment between January 2012 and December 2018. We excluded those who were transferred to long‐term care at the time of discharge. We then selected those who had filled at least 2 LLT prescriptions in the first year after CHD hospitalization, and the date of the second LLT dispensation was selected as the index date. This was to define a cohort with chronic LLT use and to be able to calculate an initial assessment of treatment adherence (Figure 1B). An overview of the longitudinal study design is presented in Figure 1A and Figure S1.

### Exposure Assessment

The use of LLT over time was ascertained through longitudinal evaluation of all pharmacy dispensations in Swedish pharmacies, which was obtained through linkage with the prescribed drug register.^14^ We extracted information on pharmacy fills with statins (*Anatomical Therapeutic Chemical* [*ATC*] code C10AA) and ezetimibe (*ATC* code C10AX09) before and after the CHD event (Table 1). We summarized the expected percentage reduction in LDL‐C for each dose of each statin, using data derived from pivotal trials (Table 1).^15^, ^16^, ^17^, ^18^ The percentage reduction with ezetimibe monotherapy of 19% classifies it as low intensity. When ezetimibe was combined with a statin we calculated the additional LDL‐C reduction on top of the percent reduction with statin therapy.^3^, ^7^ We did not consider PCSK9 (proprotein convertase subtilisin/kexin type 9) inhibitors in our analysis, because they were rarely prescribed. For each dispensation, the expected percent reduction in absolute LDL‐C is defined as its intensity.

**Table 1:** Classification of Treatment Intensity and Lipid‐Lowering Therapy–Mediated Expected Percent Reduction of Low‐Density Lipoprotein Cholesterol With Different Dosages as Observed in Clinical Trials

Statin	Low intensity	Moderate intensity	High intensity	*ATC* code
Simvastatin	10 mg	20 mg, 40 mg		C10AA01
Atorvastatin	…	10 mg, 20 mg	40 mg, 80 mg	C10AA05/C10BX03
Fluvastatin	20 mg, 40 mg	80 mg	…	C10AA04
Pravastatin	10 mg, 20 mg	40 mg	…	C10AA03/C10BX02
Rosuvastatin	…	5 mg, 10 mg	20 mg, 40 mg	C10AA07

Therapy	Dose, mg/d					Reference
	5	10	20	40	80	
Fluvastatin			21%*	27%*	33%†	43
Pravastatin		20%*	24%*	29%†		
Simvastatin		27%*	32%†	37%†	42%†	
Atorvastatin		37%†	43%†	49%‡	55%‡	
Rosuvastatin	38%†	43%†	48%‡	53%‡	58%‡	
Ezetimibe monotherapy		19%*				17, 18
Ezetimibe added to statin		24%§				3, 17

Data are presented as the relative (percent) reductions in low‐density lipoprotein cholesterol. ATC indicates Anatomical Therapeutic Chemical.

* Low intensity.

^†^ Moderate intensity.

^‡^ High intensity.

^§^ Varies by statin: 24% reduction for ezetimibe applied to remaining low‐density lipoprotein after applying statin percent reduction.

Traditional adherence analyses are based on specific time periods (eg, adherence during the first year of therapy), which are conditional on surviving and being on treatment. In diseases with high mortality risks, this can lead to underestimation of effects and immortal time bias. To minimize this, we considered each LLT dispensation as a time‐dependent exposure and created a rolling assessment,^19^, ^20^ updated at each new dispensation, of the estimated adherence and average intensity in each previous 12‐month period. The average intensity was based on the proportion of months in the prior year spent on different intensities. Thus, if a patient was 8 months on 80 mg of atorvastatin (expected LDL‐C reduction of 55%) and 4 months on 40 mg simvastatin (expected LDL‐C reduction of 37%), the average LLT intensity in that year was 49%. Based on the expected mean LDL‐C reductions from trials, this metric ranges from 0% to 66% and was further categorized as low‐moderate intensity and high‐intensity LLT according to Table 1. Similarly, we measured adherence in the year before each LLT dispensation using the proportion of days covered (PDC) method.^20^ This metric ranges from 0% to 100% adherence, and patients were categorized at each pharmacy fill as adherent (PDC≥0.80), poorly adherent (PDC<0.80), and discontinuers (PDC=0) (Figure S2). When patients had <12 months of observation on therapy, we calculated their average adherence and intensity since the index date (Figure 1A).

Previous research has examined the drawbacks of dichotomizing PDC into a binary measure.^21^, ^22^ We created a combined measure of LLT adherence‐adjusted intensity by multiplying the PDC by the average LLT intensity at each pharmacy fill, as proposed by Khunti et al.^11^ This combined measure is a continuous variable that is statistically more robust^22^, ^23^ and allows estimates in patients who discontinue treatment.

### Covariate Assessment

Study covariates were assessed at baseline as well as at each new LLT dispensation, and included age, sex, laboratory measurements (creatinine, blood lipids), medical history, ongoing medications, recent health care use, days from CHD discharge to the first LLT dispensation, and calendar year. Detailed definitions for these covariates are shown in Table S1. Outpatient creatinine and LDL‐C levels were assessed on or before the first dispensation date after the CHD hospitalization. Creatinine was used to calculate the estimated glomerular filtration rate using the 2009 Chronic Kidney Disease Epidemiology Collaboration equation^24^ without correction for race. Including LDL‐C levels as a study covariate is important to reduce confounding; high LDL‐C levels may motivate decisions to be switched to more intense LLT or to be more adherent to therapy.

### Study Outcomes

The primary outcome was the occurrence of MACE, a composite of nonfatal myocardial infarction, nonfatal ischemic stroke, and all‐cause death (Table S2). The main secondary outcome was the attainment of the guideline‐recommended LDL‐C goal of <1.8 mmol/L^25^, ^26^ (70 mg/dL), which was calculated from the outpatient LDL‐C measurement closest to each statin fill. Other secondary outcomes included the individual components of MACE, hospitalization for heart failure, and hospitalization for unstable angina. Time at risk began after the index date (ie, the date of the second LLT dispensation) and stopped upon the first occurrence of a study outcome, a switch to or addition of a PCSK9 inhibitor, death, migration, or end of follow‐up (December 31, 2019), whichever occurred first.

### Statistical Analysis

Demographic and clinical characteristics of the patients are presented as number and percentage for categorical variables and mean and SD, median and interquartile range, and range for continuous variables.

We used multivariable Cox proportional hazard regression with time‐varying covariates to evaluate the associations between 1‐year treatment intensity, 1‐year adherence, and their combined product (ie, adherence‐adjusted intensity) with the risk of study outcomes. Each continuous measure was divided by 10 so that each 1‐unit change in the β estimate was attributed to a 10% absolute change in continuous measures. We evaluated these exposures as continuous and categorical variables in 3 separate models, presenting adjusted hazard ratios (HRs) with 95% CIs. We evaluated the consistency of our findings through prespecified subgroup analyses, which included sex, age (<65 and ≥65 years), CHD type (revascularization/acute myocardial infarction), diabetes, estimated glomerular filtration rate categories (<60 and ≥60 mL/min per 1.73 m^2^), history of previous CHD, baseline LDL‐C of ≥3.5 mmol/L, and prevalent LLT use to estimate multiplicative interactions by the likelihood ratio test.

We used mixed multivariable logistic regression that adjusts for within‐subject correlation. The model includes subjects as random effects and a fixed effect for patients, to estimate the odds ratios (ORs) for the attainment of LDL‐C goals over time. We formally tested for continuous‐by‐continuous interaction between continuous measures of adherence and treatment intensity to illustrate the relationship between the 2 measures and the probability of LDL‐C goal attainment. Data were analyzed using R version 4.0.5 (R Foundation for Statistical Computing).

### Sensitivity Analyses

The assumption underlying the exposure intensity measure is that patients who discontinue treatment receive 0 intensity in that year, and thus the intensity measure is directly tied to the adherence measure. We performed a sensitivity analysis in which patients were censored when discontinuing treatment to analyze only patients on treatment.

## RESULTS

Figure 1B shows the patient selection flow for our study, resulting in 20 490 eligible patients who survived a CHD admission, initiated or continued LLT treatment, and received a minimum of 2 consecutive dispensations. The median (interquartile range) time between hospital discharge and a second dispensation of LLT was 91 days (71–111 days). The majority initiated high‐intensity LLT (62%), and the remaining 38% initiated low‐moderate–intensity LLT. Ezetimibe monotherapy was rare (n=62), as were combinations of a statin with ezetimibe (n=1012, 5%) at baseline. Statin+ezetimibe combinations increased to 17% during the period of observation, but only 98 patients were initiated on a PCSK9 inhibitor. The demographic and clinical characteristics of the patients are presented in Table 2 and Table S3. Their average age was 68±11 years, and 25% were women. The most frequent comorbidity was hypertension (72%), followed by a history of CHD (33%) (composite of myocardial infarction, angina, and other ischemic heart diseases), diabetes (29%), congestive heart failure (25%), and chronic kidney disease, defined as estimated glomerular filtration rate <60 mL/min per 1.73 m^2^ (19%). Nearly all patients in the study were treated with antiplatelet agents (99%) and a β‐blocker (91%).

**Table 2:** Baseline Characteristics of the Cohort

Characteristic	Value, overall, N=20 490
CHD qualifying event type, n (%)	
Acute myocardial infarction	13 879 (68%)
Stable CHD receiving revascularization	6661 (32%)
Age, y, mean (SD)	68.1 (11.2)
Age categories, y, n (%)	
18–49	1112 (5%)
50–64	6263 (31%)
65–79	9903 (48%)
≥80	3212 (16%)
Sex, women, n (%)	5122 (25%)
LDL cholesterol, mmol/L, mean (SD), n=20 002	2.26 (0.95)
Total cholesterol, mmol/L, mean (SD), n=18 837	4.11 (1.12)
HDL cholesterol, mmol/L, mean (SD), n=18 555	1.23 (0.38)
Triglycerides, mmol/L, mean (SD), n=18 599	1.45 (0.84)
eGFR, mL/min per 1.73 m^2^, mean (SD), n=20 122	76.0 (20.0)
eGFR, mL/min per 1.73 m^2^, categories	
≥60	16 188 (79%)
30–59	3476 (17%)
<30	458 (2%)
Missing	368 (2%)
Comorbidities, n (%)	
CVD, composite	6709 (33%)
History of myocardial infarction	2858 (14%)
History of revascularization	3254 (16%)
Diabetes	5909 (29%)
Hypertension	14 791 (72%)
Heart failure	5125 (25%)
Peripheral artery disease	2129 (10%)
Stroke	1504 (7%)
Transient ischemic attack	929 (4%)
Medications, n (%)	
Antiplatelets	20 214 (99%)
β‐Blockers	18 738 (91%)
Nitrates	17 673 (86%)
Renin‐angiotensin‐system inhibitors	16 538 (81%)
LLT before event	10 336 (50%)
Calcium channel blockers	6893 (34%)
Diuretics	6379 (31%)
Anticoagulants	3142 (15%)
Digoxin	398 (2%)
LLT intensity at index, n (%)	
High	12 740 (62.2%)
Low‐moderate	7750 (37.8%)
Ezetimibe monotherapy	62 (0.3%)
Statins with ezetimibe	1012 (5%)
Adherence to LLT at index, n (%)	
Poorly adherent, PDC<0.8	1795 (9%)
Adherent, PDC≥0.80	18 695 (91%)

Additional characteristics are described in Table S4. CHD indicates coronary heart disease; CVD, cardiovascular disease; eGFR, estimated glomerular filtration rate; HDL‐C, high‐density lipoprotein cholesterol; LDL‐C, low‐density lipoprotein cholesterol; LLT, lipid‐lowering therapy; and PDC, proportion of days covered.

The patients were followed for a total of 72 839 patient‐years, with a mean of 2.6±1.1 years, ranging from 1 to 7 years. During that period, 279 885 LLT dispensations were recorded (Figure S3). The median (interquartile range) number of dispensations per person was 13 (8–19), and the range was 4 to 52. At baseline, 91% of patients were adherent (PDC≥80%). This proportion decreased gradually over time but remained high, with 70% of patients being considered adherent to therapy after 7 years. Only 2% of the patients discontinued treatment permanently (Tables S4 and S5). A total of 19 374 subjects had at least 1 LDL‐C measurement after the index date, with a total of 58 397 LDL‐C measurements during the period of observation. The median (range) number of LDL‐C measurements per patient was 2 (1–16).

### Associations of Adherence and Treatment Intensity With the Risk of MACE

**Figure 2:**
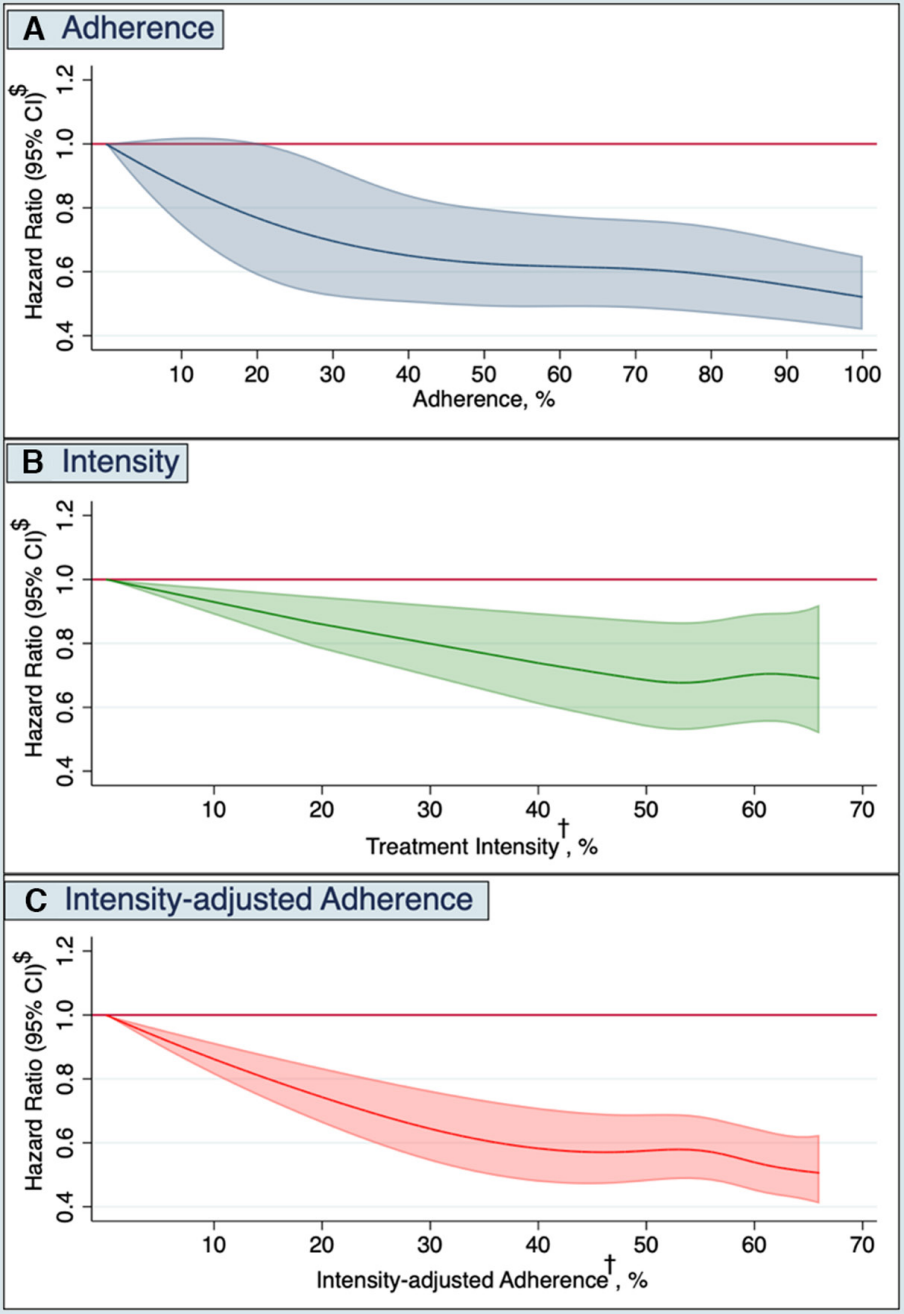
Graphical representation of the associations between continuous measures of statin (**A**) adherence, (**B**) intensity, or (**C**) adherence‐adjusted intensity, and the risk of MACE. †The exposure statin intensity ranges from 0% to 66%, given that the highest doses of the most potent lipid‐lowering therapy are estimated to lower low‐density lipoprotein cholesterol (LDL‐C) by 66% in clinical trials (see Table 1). The exposure of adherence ranges from 0% (nonuse) to 100%. The combined exposure of adherence‐adjusted intensity is the product between the 2, thus ranging from 0% to 66%. $Models adjusted for demographics (ie, age, sex), estimated glomerular filtration rate, LDL‐C, number of LDL‐C measurements, all comorbidities (history of previous myocardial infarction, previous revascularization, diabetes, hypertension, heart failure, peripheral artery disease, valve disorder, stroke, transient ischemic attack, atrial fibrillation, other arrhythmias, chronic respiratory disease, other lung diseases, venous thromboembolism, liver disease, cancer, fracture in previous year), medications (β‐blockers, calcium channel blockers, diuretics, renin‐angiotensin‐system inhibitors, digoxin, nitrates, antiplatelet, anticoagulants, β‐2 agonist, anticholinergic inhalants, glucocorticoids, inhalants, oral glucocorticoids, NSAIDs, opioids), health care use (cardiovascular hospitalizations in the previous year, noncardiovascular hospitalizations during the past year, outpatient contacts for cardiovascular causes during the past year, outpatient contacts for noncardiovascular reasons in previous year, number of unique dispensed drugs during the past year), calendar year, and education level.

A total of 3046 patients (15%) experienced MACE, with a mean event rate of 38 per 1000 person‐years (Tables S6 and S7). Figures 2A through 2C show the associations between the continuous measures of adherence, treatment intensity, and adherence‐adjusted intensity with the risk of suffering MACE.

Each 10% increment in adherence was associated with a 6% reduction in risk of MACE (HR, 0.94 [95% CI, 0.93–0.96]). This was attributed to risk reductions in all individual MACE components (Table S8). In addition, each 10% increment in adherence was associated with a lower risk of unstable angina (HR, 0.97 [95% CI, 0.95–0.99]), and a tendency toward a modest reduction of the risk of heart failure (HR, 0.98 [95% CI, 0.95–1.00]) (Table S9). When adherence was analyzed as a categorical exposure and compared with patients who were adherent (PDC≥80%), poor adherence (PDC<80%) and discontinuation of LLT were associated with higher risks of MACE (HR, 1.23 [95% CI, 1.12–1.34] and HR, 1.66 [95% CI, 1.23–2.22], respectively) (Table 3). Associations with the individual components of MACE as well as with other secondary CVD outcomes were generally in the same direction (Table S9).

**Table 3:** Associations of Categories of Statin Adherence, Intensity, and Adherence‐Adjusted Intensity With the Risk of MACE and With the Attainment of LDL‐C Goals

Category	Risk of suffering MACE,* aHR (95% CI)	Odds of reaching LDL‐C goals,† OR (95% CI)
Statin adherence		
Adherent, PDC≥80%	REF	REF
Poorly adherent	1.23 (1.12–1.34)	0.67 (0.60–0.75)
Discontinued ≥1 y	1.66 (1.23–2.22)	0.20 (0.13–0.30)
Statin intensity		
High intensity, ≥50% LDL reduction	REF	REF
Low‐moderate intensity, <50% LDL reduction	1.00 (0.92–1.08)	0.71 (0.64–0.78)
Discontinued ≥1 y	1.57 (1.17–2.10)	0.14 (0.09–0.21)
Combined adherence and treatment intensity		
High intensity, adherent	REF	REF
Low‐moderate intensity, adherent	0.96 (0.88–1.05)	0.70 (0.63–0.79)
Low‐moderate intensity, poorly adherent	1.32 (1.17–1.48)	0.54 (0.45–0.64)
High intensity, poorly adherent	1.16 (1.01–1.33)	0.69 (0.61–0.79)

Adherent patients are those with a proportion of days covered of 80% or higher for the year. 1.8 mmol/L LDL‐C=70 mg/dL. aHR indicates adjusted hazard ratio; LDL, low‐density lipoprotein; LDL‐C, low‐density lipoprotein cholesterol; MACE, major adverse cardiovascular events; OR, odds ratio; PDC, proportion of days covered; and REF, reference.

* Output from mixed‐effects logistic regression models. Models adjusted for demographics (ie, age, sex), estimated glomerular filtration rate, LDL‐C (only in Cox model), average number of LDL‐C measurements, all comorbidities (history of previous myocardial infarction, previous revascularization, diabetes, hypertension, heart failure, peripheral artery disease, valve disorder, stroke, transient ischemic attack, atrial fibrillation, other arrhythmias, chronic respiratory disease, other lung diseases, venous thromboembolism, liver disease, cancer, fracture in previous year, medications (β‐blockers, calcium channel blockers, diuretics, renin‐angiotensin‐system inhibitors, digoxin, nitrates, antiplatelet, anticoagulants, β‐2 agonist, anticholinergic inhalants, glucocorticoids, inhalants, oral glucocorticoids, NSAIDs, opioids), health care use (cardiovascular hospitalizations in the previous year, noncardiovascular hospitalizations during the past year, outpatient contacts for cardiovascular causes during the past year, outpatient contacts for noncardiovascular reasons in previous year, number of unique dispensed drugs during the past year), calendar year, and education level.

^†^ Output from Cox regression models.

Similarly, every 10% increase in treatment intensity was associated with an 8% reduction in the risk of MACE (HR, 0.92 [95% CI, 0.88–0.96]). As a categorical exposure, and compared with high‐intensity LLT, patients on low‐moderate–intensity treatment had a similar MACE risk (HR, 1.00 [95% CI, 0.92–1.08]), whereas patients who discontinued LLT were at a higher MACE risk (HR, 1.57 [95% CI, 1.17–2.10]) (Table 3). Results were consistent when assessing the individual components of MACE (Table S10). In addition, patients with low‐moderate–intensity LLT, compared with those with high‐intensity LLT, had modest effects on heart failure and unstable angina (Table S10).

When modeling the combined measure of adherence‐adjusted intensity, each 10% increase was associated with a lower risk of MACE by 9% (HR, 0.91 [95% CI, 0.89–0.94]), and this was also observed for secondary outcomes (Table S10). Adherent patients had the same MACE risk whether they were treated with low‐moderate or high‐intensity LLT (Table 3). Patients who were poorly adherent and received low‐moderate–intensity LLT had a higher MACE risk (HR, 1.32 [95% CI, 1.17–1.48]) and mortality risk (HR, 1.55 [95% CI, 1.34–1.77]) than adherent patients who received high‐intensity treatment (Table 3 and Table S10). Similarly, poorly adherent patients receiving a high‐intensity regimen had a 16% higher MACE risk (HR, 1.16 [95% CI, 1.01–1.33]) than their adherent counterparts.

**Figure 3:**
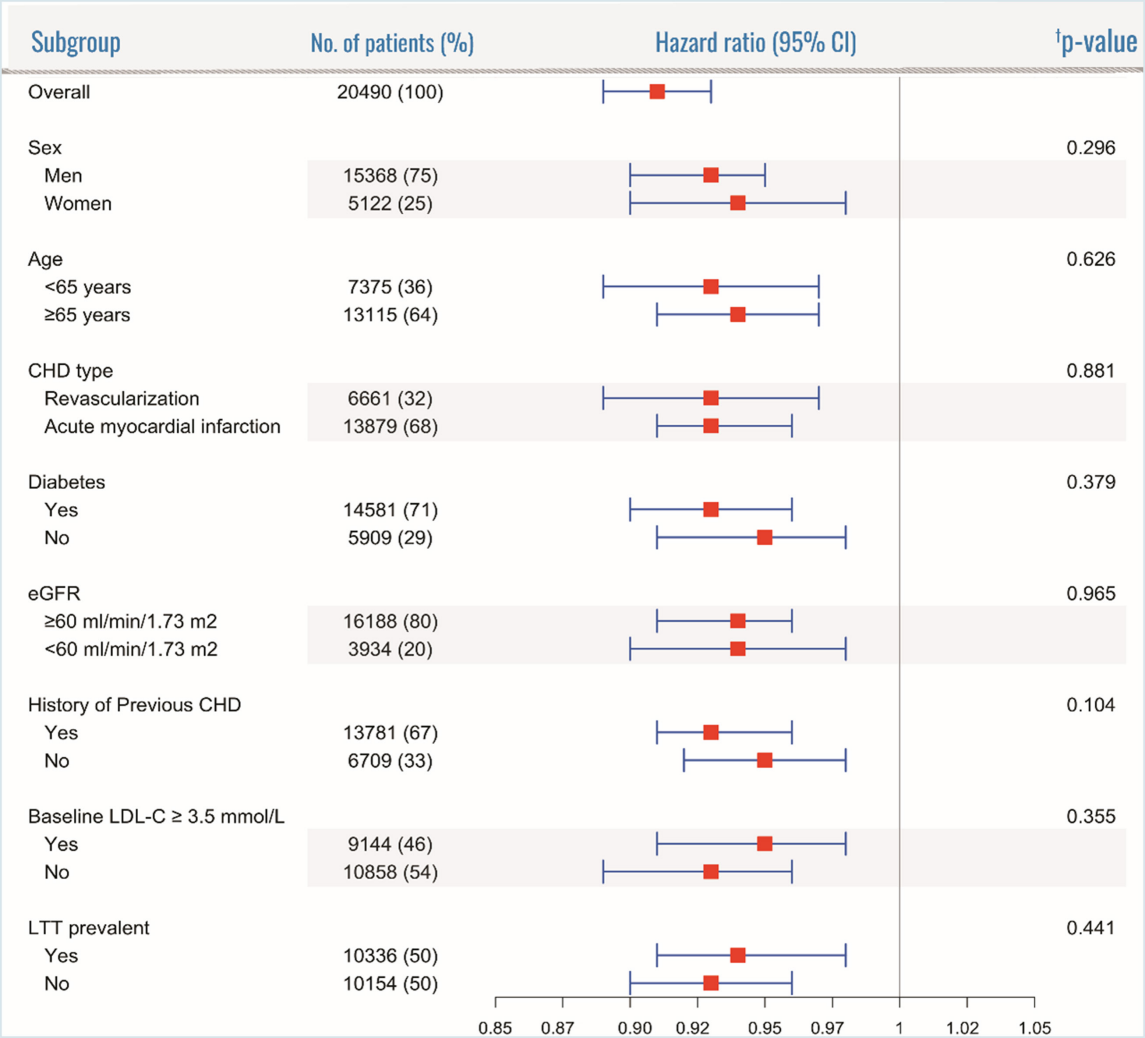
Cardiovascular risk reduction associated with each 10% increment in adherence‐adjusted intensity across subgroups. CHD indicates coronary heart disease; eGFR, estimated glomerular filtration rate; LDL‐C, low‐density lipoprotein cholesterol; and LLT, lipid‐lowering therapy. †The *P* value is from the test statistic for testing interaction between the exposure and any subgroup variable. Models adjusted for demographics (ie, age, sex), eGFR, LDL‐C, average number of LDL‐C measurements, all comorbidities (history of previous myocardial infarction, previous revascularization, diabetes, hypertension, heart failure, peripheral artery disease, valve disorder, stroke, transient ischemic attack, atrial fibrillation, other arrhythmias, chronic respiratory disease, other lung diseases, venous thromboembolism, liver disease, cancer, fracture in previous year), medications (β‐blockers, calcium channel blockers, diuretics, renin‐angiotensin‐system inhibitors, digoxin, nitrates, antiplatelet, anticoagulants, β‐2 agonist, anticholinergic inhalants, glucocorticoids, inhalants, oral glucocorticoids, NSAIDs, opioids), health care use (cardiovascular hospitalizations in the previous year, noncardiovascular hospitalizations during the past year, outpatient contacts for cardiovascular causes during the past year, outpatient contacts for noncardiovascular reasons in previous year, number of unique dispensed drugs during the past year), calendar year, and education level.

Results were consistent across subgroups evaluated (Figure 3), and the magnitude of the associated risk reduction was thus higher among high‐risk subgroups.

### Associations of Adherence and Treatment Intensity with LDL‐C Goal Attainment

**Figure 4:**
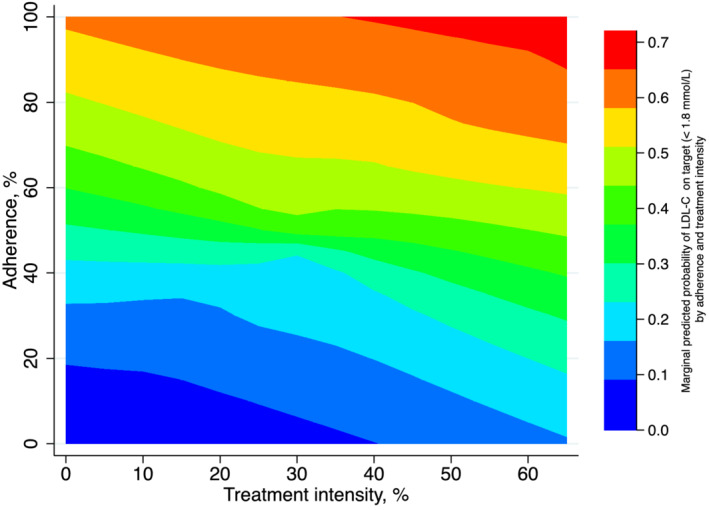
The effect of adherence (*y* axis, %) on the predicted probability of low‐density lipoprotein cholesterol (LDL‐C) goal attainment across levels of intensity (*x* axis, %) and vice versa. The exposure treatment intensity ranges from 0% to 66%, given that the highest doses of the most potent lipid‐lowering therapy are estimated to lower LDL‐C by 66% in clinical trials (see Table 1). The exposure of treatment adherence ranges from 0% (nonuse) to 100%. 1.8 mmol/L LDL‐C=70 mg/dL. Results of repeated‐measures logistic regression with an interaction between 2 continuous covariates. The model includes subjects as random effects and fixed effects for patient demographics (ie, age, sex), estimated glomerular filtration rate, average number of LDL‐C measurements, all comorbidities (history of previous myocardial infarction, previous revascularization, diabetes, hypertension, heart failure, peripheral artery disease, valve disorder, stroke, transient ischemic attack, atrial fibrillation, other arrhythmias, chronic respiratory disease, other lung diseases, venous thromboembolism, liver disease, cancer, fracture in previous year), medications (β‐blockers, calcium channel blockers, diuretics, renin‐angiotensin‐system inhibitors, digoxin, nitrates, antiplatelet, anticoagulants, β‐2 agonist, anticholinergic inhalants, glucocorticoids, inhalants, oral glucocorticoids, NSAIDs, opioids), health care use (cardiovascular hospitalizations in the previous year, noncardiovascular hospitalizations during the past year, outpatient contacts for cardiovascular causes during the past year, outpatient contacts for noncardiovascular reasons in previous year, number of unique dispensed drugs during the past year), calendar year, and education level.

Each 10% increase in adherence (OR, 1.12 [95% CI, 1.10–1.15]), intensity (OR, 1.42 [95% CI, 1.34–1.51]), or the product term of adherence and intensity (OR, 1.16 [95% CI, 1.19–1.24]) was associated with higher odds LDL‐C goal attainment (Table 3, right column). Patients with adherence <80% had a higher risk of not achieving LDL<1.8 mmol/L compared with adherent patients. A similar pattern was observed for low‐moderate versus high intensity. Patients who were poorly adherent and received low‐moderate–intensity LLT had lower odds of LDL‐C goal attainment than patients who received high‐intensity treatment and had optimal adherence. Figure 4 illustrates the effect of treatment intensity on the predicted probability of having LDL‐C on target across levels of adherence and vice versa.

### Sensitivity Analyses

Restricting our analyses to patients who did not discontinue therapy provided similar results, both in magnitude and direction, as our main analysis (Table S11, Figure S4A through S4C and Figure S5).

## DISCUSSION

By observing all patients who were hospitalized for CHD and subsequently initiated or continued LLT in the Stockholm region during 2012 to 2018, we made the following findings. Good adherence to high‐intensity LLT was associated with increased odds of LDL‐C goal attainment and the lowest hazard of MACE and death from all causes. With regard to MACE, good adherence appeared to be more important than treatment intensity. Our results were robust across sensitivity analyses, subgroups, and secondary outcomes. Collectively, these findings show the need to implement strategies that improve adherence and greater use of intensive therapies in secondary CVD prevention, because this could substantially reduce cardiovascular risk.

Despite well‐established cause‐and‐effect relationships between LDL‐C and CVD, the inertia of prescribing and titrating statins persists, which can adversely affect the quality of care in secondary prevention.^5^ Our data align with results from trials^1^, ^27^ and previous observational studies,^11^, ^12^, ^28^ showing that the use of more intensive therapies or higher medication adherence are, when considered separately, associated with better clinical outcomes. In our study, ≈60% of the patients were on high‐intensity treatment, which is a greater proportion than reported in other health care systems^11^, ^12^, ^29^ but far from optimal. We observed a rate of adherence that was superior to other health care systems,^11^, ^12^, ^29^, ^30^ but we also showed that adherence was highest during the first year of treatment and decreased progressively over time. This is important to emphasize, because previous studies have primarily studied early adherence^28^ (ie, when patients suffering a CHD event presumably are the most motivated to follow recommendations). Reasons for the progressive decline in LLT adherence are not well‐known, but may be attributed to patient‐related factors such as age, socioeconomic factors,^31^ drug intolerance, adverse effects, or comorbidities.^32^

In keeping with previous studies,^8^, ^33^, ^34^, ^35^, ^36^, ^37^ we observed unfavorable outcomes for people discontinuing their LLT. However, we feel that the interpretation of this result is complex, because it may represent a biological rebound or a risk‐treatment mismatch phenomenon, where treatment is withdrawn from severely ill patients or patients with intolerance/adverse effects.

A novel contribution of our study is the evaluation of a combined measure of adherence and treatment intensity, allowing us to explore the complex interplay between these aspects of drug treatment. We observed that greater adherence‐adjusted intensity of LLT was associated with a greater LDL‐C goal attainment and a lower risk of MACE. Of interest, high‐ compared with low‐moderate–intensity treatment was associated with increased odds of attaining the LDL‐C goal of <1.8 mmol/L, but the risk of MACE did not differ between the 2 groups. Thus, although the 2 aspects of LLT management interact with each other, adherence was a stronger determinant of the observed clinical benefit than treatment intensity in our study. Other researchers have also found that, after controlling for statin intensity, better adherence leads to better post‐AMI outcomes.^38^, ^39^ Thus, we speculate that it may be more important to be adherent to LLT than to achieve a small further LDL‐C reduction through increased LLT intensity.

Our findings expand to routine care the previous results from clinical trials,^40^, ^41^ and largely agree with 3 observational studies that evaluated individuals attending primary health care consultations or patients with a history of myocardial infarction.^12^, ^28^ Compared with these studies, our analysis has several strengths. We included a contemporary CHD population with a long follow‐up, and our setting in the context of universal health care access is less affected by health care access bias or financial constraints to purchase medications than in the previous studies. The complete capture of health care (primary, secondary, and hospital care) allows the evaluation of the collective care of all inhabitants in the region and is not affected by health care fragmentation. By focusing on medication use after CHD, we minimize biases derived from evaluating long‐term survivors. We used pharmacy dispensations rather than prescriptions, which reduces primary noncompliance bias. Of note, some 20% of the eligible patients did not claim LLT within 12 months after hospital discharge. Our roll‐out design at each dispensation is a methodological improvement that not only minimizes the immortal time bias of evaluating treatment adherence within fixed periods, but also has benefits over landmark analyses to alleviate selection bias. Finally, by including laboratory information such as estimated glomerular filtration rate and LDL‐C, we are able to improve our control of confounding. Specifically, regular LDL‐C monitoring may indicate health‐seeker or adherent behaviors,^42^ and persistently high or low LDL‐C informs clinical decisions on the need to improve adherence or to change treatment.

Our study also has limitations. Our results apply to patients with CHD in Stockholm, Sweden, during 2012 to 2019. Extrapolation to other health care systems or periods should be done with caution. As with any observational study, a causal interpretation of the observed associations also requires caution. Treatment adherence may be related to factors that are not quantifiable in this analysis, such as lifestyle modifications, drug intolerance, or adverse effects. Likewise, it is possible that part of the protective effect could be attributed to a healthy user effect, because subjects with a high degree of adherence may also have a healthier behavior in general, such as adherence to other cardiovascular medications or adherence to healthy life‐style advice.^43^ As shown in a meta‐analysis, poor adherence to single or combined cardiovascular pharmacotherapies significantly increases the incidence of adverse cardiovascular events and death.^44^ Finally, statistical significance (or a CI that does not cross the null value of 1) may have occurred by chance alone because of the high number of tests performed.

To conclude, in this evaluation of patients with CHD undergoing routine care, adherence to LLT was associated with the greatest benefit in terms of secondary cardiovascular prevention regardless of treatment intensity. LDL‐C goal attainment was, however, improved by high‐intensity treatment. Numerous strategies to improve statin treatment in patients with hypercholesterolemia have been used, with varying degrees of success. Although these generally short‐term interventions improve statin prescribing and statin adherence, no single strategy or group of strategies has consistently improved outcomes.^45^ The best strategy is likely both multifaceted and tailored to the individual. Providing person‐centered secondary prevention care is important, given that this care would be respectful to individual patient values and facilitate patient discussions conveying risk and encouraging treatment adherence in the long term.^27^, ^46^

## Sources of Funding

This study was funded by the Swedish Research Council (number 2019–01059), the Swedish Heart and Lung Foundation (number 20190587), and the Westman Foundation.

## Disclosures

J.J.C. received institutional funding from AstraZeneca, Astellas, Amgen, and ViforPharma outside this study. C.M.C. received consultation, advisory board membership, honoraria, or research funding from the Ontario Ministry of Health, Sanofi, Pfizer, Leo Pharma, Astellas, Janssen, Amgen, Boehringer‐Ingelheim, and Baxter, and through LiV Academy and AstraZeneca. T.J. received institutional funding from Novartis outside this study. The remaining authors have no disclosures to report.

## Supporting information

Data S1
Tables S1–S11
Figures S1–S5
