# Supplementary material for: Intensity of and Adherence to Lipid‐Lowering Therapy as Predictors of Major Adverse Cardiovascular Outcomes in Patients With Coronary Heart Disease
Source: J Am Heart Assoc. 2022 Jul 5;11(14):e025813. doi: 10.1161/JAHA.122.025813 (PMC9707817; doi:10.1161/JAHA.122.025813)
Supplement: Supplementary file 1 — Data S1 Tables S1–S11 Figures S1–S5 [file JAH3-11-e025813-s001.pdf]

# SUPPLEMENTAL MATERIAL

**Data S1.**

## Supplemental Methods

### Case definition of coronary heart disease (CHD) event

Hospitalizations with acute myocardial infarction (AMI), PCI or CABG from 2012 to 2018 as ascertained by the ICD-10 codes and/or the Nordic Medico-Statistical Committee (NOMESCO) classification codes issued at discharge. AMI was defined by hospital discharge with ICD-10 code I21, PCI or CABG were ascertained by coronary revascularization procedural codes FNA-H, FNJ-K, FNW.

**Table S1. Definitions and categorizations of covariates.**

| Variable | Categorization | ICD 10 codes |
|---|---|---|
| ***Sociodemographic*** | | |
| Sex | Men; Women | |
| Age category, years | 18-49; 50-64; 65-79; ≥80 | |
| ***Highest attained education*** | | |
| Compulsory school | Yes/No | |
| Secondary school | Yes/No | |
| University | Yes/No | |
| ***Laboratory variables*** | | |
| eGFR (mL/min/1.73 m2) | Continuous | |
| eGFR category (mL/min/1.73 m2) | >60 / 60 - 29 / <30 | |
| LDL-C | Continuous | |
| Average number of LDL-C measurements | Continuous | |
| ***History of cardiovascular events*** | Yes/No | Previous CHD (I20, I21, I22, I25) |
| ***Comorbidities*** | | |
| Acute coronary syndrome | Yes/No | I200, I21-I22 |
| Other ischemic heart disease | Yes/No | I201, I208, I209, I24, I25 |
| Hypertension | Yes/No | I10-I15 |
| Diabetic Mellitus | Yes/No | E10 – E14 |
| Heart failure | Yes/No | I110, I130, I132, I50 |
| Valve disorders | Yes/No | I34-I37 |
| Stroke | Yes/No | I61, I63, I64 |
| TIA | | G450, G451, G452,G453, G458, G459 |
| Other cerebrovascular disease | Yes/No | I65-I69, G46 |
| Atrial fibrillation | Yes/No | I48 |
| Other arrhythmia | Yes/No | I44-I47, I49 |
| Peripheral vascular disease | Yes/No | I70, I72, I73 |
| Chronic resp disease | Yes/No | J40-J47 |
| Other lung disease | Yes/No | I270, I272, I278, I279, J61-J69, J70, J84, J92, J96, J982, J983 |

| | | |
|---|---|---|
| Venous thromboembolism | Yes/No | I26, I80 (excl I80.0), I81, I820, I822-I829 |
| Cancer in previous year | Yes/No | C00-C43, C45-C97 |
| Liver disease | Yes/No | B18, I850, I859, I982, K70-K77 |
| Fracture in previous year | Yes/No | S02 (except S025), S12, S22, S32, S42, S52, S62, S72, S82, S92, T02, T08, T10, T12, M484, M485, M843 |
| ***Medications*** | | **ATC codes** |
| Beta blockers | Yes/No | C07 |
| Calcium-channel blockers | Yes/No | C08 |
| Diuretic | Yes/No | C03 |
| ACEi/ARB | Yes/No | C09A, C09B, C09C, C09D |
| Digoxin | Yes/No | C01AA05 |
| Nitrate | Yes/No | C01DA |
| Antiplatelet | Yes/No | B01AC |
| Anticoagulant | Yes/No | B01AA, B01AE07, B01AF, B01AX05 |
| β-2 agonist inhalant | Yes/No | R03AC |
| Anticholinergic inhalant | Yes/No | R03BB |
| Glucocorticoid inhalant | Yes/No | R03BA, R03AK |
| Oral glucocorticoid | Yes/No | H02AB |
| NSAID | Yes/No | M01A |
| Opioid | Yes/No | N02A |
| **No of dispensed drugs in previous year** | | |
| 0-5 | | |
| 6-10 | | |
| 11-15 | | |
| >15 | | |
| **Calender year** | | |
| 2012 | | |
| 2013 | | |
| 2014 | | |
| 2015 | | |
| 2016 | | |
| 2017 | | |
| 2018 | | |
| 2019 | | |
| **Hospitalizations in previous year or within the preceding eligibility window** | | |

| | | |
|---|---|---|
| Number of hospitalizations during previous year Cardiovascular causes | Yes/No | All hospitalizations recorded in the National Patient Registry in the previous year I00-I99 (primary position) |
| Non-cardiovascular related causes | Yes/No | Not I00-I99 (primary position) |
| **Outpatient contacts in previous year or within the preceding eligibility window** | | |
| Number of outpatient contacts during previous year | Yes/No | I00-I99 (primary position) |
| Cardiovascular causes | Yes/No | E11 (primary position) |
| Non-cardiovascular related causes | | I00-I99 (primary position) |

**Table S2. Definition for study outcomes.**

| | Coding System | Codes |
|---|---|---|
| **Primary outcome; Major adverse cardiac events (MACE) composite of:** | | |
| Myocardial infarction | ICD-10 | I21, I22, I23 |
| Ischemic stroke | ICD-10 | H341, G45-46, I60-I61, I63, I64 |
| All-cause mortality | ICD-10 | Any code |
| **Secondary outcomes** | | |
| Heart failure | ICD-10 | I110, I130, I132, I50 |
| hospitalization for unstable angina | ICD-10 | I20 |
| LDL-C < 1.8mmol/L | - | - |

ICD-10: International Classification of Diseases (ICD) $10^{th}$ revision

**Table S3. Additional Baseline Characteristics of the Cohort beyond those of Main Table 1.**

| | Overall (N=20,490) |
|---|---|
| **Comorbidities,** *n (%)* | |
| Valve disorder | 1698 (8%) |
| other CVD | 1488 (7%) |
| Atrial fibrillation | 3632 (18%) |
| Other arrythmias | 3206 (16%) |
| Chronic respiratory disease | 3611 (18%) |
| Other lung disease | 567 (3%) |
| Venous thromboembolism | 1484 (7%) |
| Liver disease | 631 (3%) |
| Cancer | 2187 (11%) |
| Fracture in previous year | 4058 (20%) |
| **Medications,** *n (%)* | |
| Beta-2 agonist | 1816 (9%) |
| Anticholinergic inhalants | 967 (5%) |
| Glucocorticoid inhalants | 2254 (11%) |
| Oral glucocorticoids | 2055 (10%) |
| NSAIDs | 3726 (18%) |
| Opioids | 4840 (24%) |
| **Health Care utilization** | |
| Hospitalizations in previous year | |
| Cardiovascular causes, median [min, max] | 0 [0, 6] |
| Non-cardiovascular causes, median [min, max] | 0 [0, 12] |
| Outpatient contacts in previous year | |
| Cardiovascular causes, median [min, max] | 0 [0, 8] |
| Non-cardiovascular causes, median [min, max] | 2.00 [0, 29] |
| No of unique dispensed drugs in previous year, *n (%)* | |
| 0-5 | 728 (4%) |
| 6-10 | 7641 (37%) |
| 11-15 | 6411 (31%) |
| >15 | 5710 (28%) |
| **Calendar Year,** *n (%)* | |
| 2012 | 2240 (11%) |
| 2013 | 2923 (14%) |
| 2014 | 3000 (15%) |
| 2015 | 2939 (14%) |
| 2016 | 2943 (14%) |
| 2017 | 2857 (14%) |
| 2018 | 2806 (14%) |

| | |
|---|---|
| 2019 | 782 (4%) |
| **Highest attained education**, *n (%)* | |
| Compulsory school | 5184 (25%) |
| Secondary school | 8618 (42%) |
| University | 6311 (31%) |
| Missing | 377 (2%) |

**Table S4. Treatment intensity over time.**

| Number of patients | Index year (N=20490) | Year 2 (N=18130) | Year 3 (N=14432) | Year 4 (N=11293) | Year 5 (N=8494) | Year 6 (N=5914) | Year 7 (N=3552) |
|---|---|---|---|---|---|---|---|
| **% of patients acc. to treatment intensity category** | | | | | | | |
| None (DC ≥ 1 year) | 0 (0%) | 256 (1.4%) | 318 (2.2%) | 270 (2.4%) | 171 (2.0%) | 106 (1.8%) | 30 (0.8%) |
| High | 12740 (62.2%) | 11359 (62.7%) | 8706 (60.3%) | 6555 (58.0%) | 4762 (56.1%) | 3120 (52.8%) | 1701 (47.9%) |
| Low-moderate | 7750 (37.8%) | 6515 (35.9%) | 5408 (37.5%) | 4468 (39.6%) | 3561 (41.9%) | 2688 (45.5%) | 1821 (51.3%) |
| **Treatment intensity** | | | | | | | |
| Mean (SD) | 58.3 (5.76) | 58.0 (8.99) | 57.2 (10.2) | 56.8 (10.4) | 56.8 (9.71) | 56.6 (9.40) | 56.6 (7.70) |
| Median [Min, Max] | 61.0 [19.0, 66] | 61.0 [0, 66] | 61.0 [0, 66] | 61.0 [0, 66] | 60.0 [0, 66] | 60.0 [0, 66] | 57.0 [0, 66] |

**Table S5. Adherence to treatment over time.**

| | Index year (N=20490) | Year 2 (N=18130) | Year 3 (N=14432) | Year 4 (N=11293) | Year 5 (N=8494) | Year 6 (N=5914) | Year 7 (N=3552) |
|---|---|---|---|---|---|---|---|
| **% of patients according to adherence level** | | | | | | | |
| None (discontinued ≥365 days) | 0 (0%) | 256 (1.4%) | 318 (2.2%) | 270 (2.4%) | 171 (2.0%) | 106 (1.8%) | 30 (0.8%) |
| Poorly adherent (PDC <0.8) | 1795 (8.8%) | 3684 (20.3%) | 2881 (20.0%) | 2140 (18.9%) | 1849 (21.8%) | 1436 (24.3%) | 1055 (29.7%) |
| Adherent (PDC ≥ 0.80) | 18695 (91.2%) | 14190 (78.3%) | 11233 (77.8%) | 8883 (78.7%) | 6474 (76.2%) | 4372 (73.9%) | 2467 (69.5%) |
| **Adherence (continuous)** | | | | | | | |
| Mean (SD) | 94.4 (11.5) | 87.3 (22.1) | 87.0 (23.3) | 86.5 (23.8) | 85.9 (24.0) | 85.2 (23.8) | 83.0 (23.7) |
| Median [Min, Max] | 100 [12.0, 100] | 99.0 [0, 100] | 100 [0, 100] | 99.0 [0, 100] | 99.0 [0, 100] | 99.0 [0, 100] | 97.0 [0, 100] |

PDC: proportion of days covered; SD: standard deviation

**Table S6. Incidence rate of Major adverse cardiovascular events by years of follow-up.**

| Follow-up year | Events | Events per 1000 person-years (95% CI) |
|---|---|---|
| | | |
| 1 | 1556 | 76.2 (72.5- 80.0) |
| 2 | 502 | 29.1 (26.7-31.8) |
| 3 | 333 | 24.5 (22.0-27.3) |
| 4 | 274 | 26.0 (23.2-29.3) |
| 5 | 195 | 25.0 (21.7-28.8) |
| 6 | 111 | 20.7 (17.2-24.9) |
| 7 | 54 | 15.0 (11.3-19.9) |
| **Total** | 3022 | 38.0 (36.7-39.4) |

**Table S7. Event counts and event rates of secondary outcomes (individual component of Major adverse cardiovascular events (MACE), heart failure and unstable angina).**

| | N | Incidence rate per 1000 person-years (95% CI) |
|---|---|---|
| MACE | 3022 | 38.0 (36.7-39.4) |
| All-cause mortality | 1924 | 23.2 (22.2-24.3) |
| Myocardial infarction | 1328 | 16.5 (15.6-17.4) |
| Ischemic stroke | 971 | 11.9 (11.2-12.7) |
| Coronary revascularization | 2306 | 30.1 (28.9-31.3) |
| Heart failure | 2233 | 28.2 (27.0-29.4) |
| Unstable angina | 2244 | 29.3 (28.1-30.5) |

**Table S8. Associations between treatment adherence and secondary study outcomes (individual component of MACE, heart failure and unstable angina).**

| | All-cause death | MI | Ischemic stroke | heart failure | Unstable angina |
|---|---|---|---|---|---|
| **Adherence** | | | | | |
| Continuous, per 10% increase | 0. 96 (0.94 - 0.98) | 0. 94 (0.91 - 0.96) | 0. 95 (0.93 - 0.98) | 0. 98 (0.95 - 1.00) | 0. 97 (0.95 - 0.99) |
| *Categorical*: | | | | | |
| Adherent; (PDC ≥ 80%) | REF | REF | REF | REF | REF |
| Poorly adherent | 1.40 (1.26- 1.56) | 1.15 (1.00- 1.35) | 1.06 (0.89- 1.26) | 1. 04 (0.92 - 1.17) | 1. 11 (0.98 - 1.25) |
| Discontinued ≥ 1 year | 1.75 (1.38- 2.23) | 3.11 (2.16- 4.48) | 2.31 (1.53- 3.50) | 1. 89 (1.28 - 2.78) | 1. 83 (1.24 - 2.69) |

Models were adjusted for demographics (i.e., age, sex), eGFR, LDL-C, average number of LDL-C measurements in the year prior, all comorbidities (history of previous mi, previous revascularization, diabetes mellitus, hypertension, heart failure, PAD, valve disorder, stroke, TIA, atrial fibrillation, other arrhythmias, chronic respiratory disease, other lung diseases, venous thromboembolism, liver disease, cancer, fracture in previous year) medications (beta-blockers, calcium channel blockers, diuretics, renin-angiotensin system inhibitors, digoxin, nitrates, antiplatelet, anticoagulants, beta-2 agonist, anticholinergic inhalants, glucocorticoid inhalants, oral glucocorticoids, NSAIDs, opioids) healthcare utilization (cardiovascular hospitalizations in the previous year, non-cardiovascular hospitalizations during the last year, outpatient contacts for cardiovascular causes during the last year, outpatient contacts for non-cardiovascular reasons in previous year, no. of unique dispensed drugs during the last year), calendar year and education level.

**Table S9. Associations between treatment intensity and secondary study outcomes (individual components of MACE, heart failure and unstable angina).**

| | All-cause death | MI | Ischemic stroke | heart failure | Unstable angina |
|---|---|---|---|---|---|
| **Statin intensity** | | | | | |
| Continuous, per 10% increase | 0. 95 (0.91 - 0.99) | 0. 88 (0.82 - 93) | 0. 90 (0.84 - 96) | 0.90 (0.86- 0.95) | 0. 94 (0.90 - 0.98) |
| *Categorical:* | | | | | |
| High-intensity; (treatment intensity ≥ 50%) | REF | REF | REF | REF | REF |
| Low-moderate intensity (treatment intensity < 50%) | 1.10 (0.98- 1.23) | 0.92 (0.81- 1.04) | 0.93 (0.80- 1.08) | 1.08 (0.98- 1.19) | 0. 99 (0.89 - 1.09) |
| Discontinued ≥ 1 year | 1.67 (1.32- 2.13) | 2.86 (1.97- 4.13) | 2.19 (1.44- 3.32) | 1.89 (1.28- 2.78) | 1. 76 (1.19 - 2.60) |

Models were adjusted for demographics (i.e., age, sex), eGFR, LDL-C, average number of LDL-C measurements in the year prior, all comorbidities (history of previous mi, previous revascularization, diabetes mellitus, hypertension, heart failure, PAD, valve disorder, stroke, TIA, atrial fibrillation, other arrhythmias, chronic respiratory disease, other lung diseases, venous thromboembolism, liver disease, cancer, fracture in previous year) medications (beta-blockers, calcium channel blockers, diuretics, renin-angiotensin system inhibitors, digoxin, nitrates, antiplatelet, anticoagulants, beta-2 agonist, anticholinergic inhalants, glucocorticoid inhalants, oral glucocorticoids, NSAIDs, opioids) healthcare utilization (cardiovascular hospitalizations in the previous year, non-cardiovascular hospitalizations during the last year, outpatient contacts for cardiovascular causes during the last year, outpatient contacts for non-cardiovascular reasons in previous year, no. of unique dispensed drugs during the last year), calendar year and education level.

**Table S10. Associations between combined adherence-treatment intensity and secondary study outcomes.**

| | All-cause death | MI | Ischemic stroke | heart failure | Unstable angina |
|---|---|---|---|---|---|
| **Combined Adherence and Treatment Intensity** | | | | | |
| Continuous, per 10% increase | 0. 92 (0.89 - 0.95) | 0. 91 (0.87 - 0.95) | 0. 94 (0.89 - 0.98) | 0. 95 (0.92 - 0.99) | 0. 96 (0.94 - 0.99) |
| Categorical | | | | | |
| High-intensity; adherent | REF | REF | REF | REF | REF |
| Low-moderate intensity, adherent | 1.06 (0.94-1.18) | 0.92 (0.80-1.05) | 0.90 (0.76-1.05) | 1.07 (0.96- 1.18) | 0.99 (0.89- 1.10) |
| Low-moderate intensity, poorly adherent | 1.55 (1.34-1.77) | 1.17 (0.94-1.44) | 1.18 (0.94-1.48) | 1.08 (0.93- 1.26) | 1.08 (0.91- 1.28) |
| High intensity, poorly adherent | 1.20 (1.00-1.45) | 1.15 (0.92-1.44) | 0.94 (0.73-1.23) | 0.97 (0.79- 1.17) | 1.14 (0.96- 1.36) |

Models were adjusted for demographics (i.e., age, sex), eGFR, LDL-C, average number of LDL-C measurements in the year prior, all comorbidities (history of previous mi, previous revascularization, diabetes mellitus, hypertension, heart failure, PAD, valve disorder, stroke, TIA, atrial fibrillation, other arrhythmias, chronic respiratory disease, other lung diseases, venous thromboembolism, liver disease, cancer, fracture in previous year) medications (beta-blockers, calcium channel blockers, diuretics, renin-angiotensin system inhibitors, digoxin, nitrates, antiplatelet, anticoagulants, beta-2 agonist, anticholinergic inhalants, glucocorticoid inhalants, oral glucocorticoids, NSAIDs, opioids) healthcare utilization (cardiovascular hospitalizations in the previous year, non-cardiovascular hospitalizations during the last year, outpatient contacts for cardiovascular causes during the last year, outpatient contacts for non-cardiovascular reasons in previous year, no. of unique dispensed drugs during the last year), calendar year and education level.

**Table S11**. **Sensitivity analysis:** Associations of statin adherence, intensity and intensity-adjusted adherence with the risk of MACE and with attainment of LDL-C goals. Output from a sensitivity analysis which censored patients at treatment discontinuation.

| | Risk of suffering MACE[a] | Odds of reaching LDL-C goals[b] |
|---|---|---|
| | **aHR (95% CI)** | **OR (95% CI)** |
| **Statin adherence** | | |
| Continuous, per 10% increase | 0. 95 (0.93 - 0.97) | 1.11 (1.08-1.13) |
| *Categorical* | | |
| Adherent (PDC ≥ 80%) | REF | REF |
| Poorly adherent | 1.20 (1.09 - 1.33) | 0.67 (0.60 - 0.75) |
| **Statin intensity** | | |
| Continuous, per 10% increase | 0.92 (0.86-0.98) | 1.51 (1.38-1.65) |
| *Categorical* | | |
| High-intensity (≥ 50% LDL reduction) | REF | REF |
| Low-moderate intensity (< 50% LDL reduction) | 1.00 (0.92- 1.09) | 0.71 (0.64- 0.78) |
| **Combined adherence and treatment intensity** | | |
| Continuous, per 10% increase | 0.93 (0.89 -0.96) | 1.12 (1.08 -1.16) |
| *Categorical* | | |
| High-intensity, adherent | REF | REF |
| Low-moderate intensity, adherent | 0.96 (0.88-1.06) | 1.09 (0.88-1.35) |
| Low-moderate intensity, poorly adherent | 1.26 (1.10-1.45) | 0.54 (0.46-0.64) |
| High intensity, poorly adherent | 1.16 (1.01-1.33) | 0.70 (0.61-0.80) |

Abbreviations: MACE = Major adverse cardiovascular events; CI: confidence interval; PDC: proportion of days covered; aHR, adjusted hazard ratio; OR, odds ratio. Adherent patients are those with a proportion of days covered of 80% or higher for the year.

[a] Output from Cox regression models; [b] Output from mixed effect logistic regression models. Models adjusted for demographics (i.e., age, sex), eGFR, LDL-C (only in Cox model), average number of LDL-C measurements in the year prior, all comorbidities (history of previous mi, previous revascularization, diabetes mellitus, hypertension, heart failure, pad, valve disorder, stroke, TIA, atrial fibrillation, other arrhythmias, chronic respiratory disease, other lung diseases, venous thromboembolism, liver disease, cancer, fracture in previous year) medications (beta-blockers, calcium channel blockers, diuretics, renin-angiotensin system inhibitors, digoxin, nitrates, antiplatelet, anticoagulants, beta-2 agonist, anticholinergic inhalants, glucocorticoids, inhalants, oral glucocorticoids, NSAIDs, opioids) healthcare utilization (cardiovascular hospitalizations in the previous year, non-cardiovascular hospitalizations during the last year, outpatient contacts for cardiovascular causes during the last year, outpatient contacts for non-cardiovascular reasons in previous year, no. of unique dispensed drugs during the last year), calendar year and education level.

ranges from 0 (non-use) to 100%. The combined exposure of intensity adjusted adherence is the product between the two, thus ranging from 0 to 66%.

[b] Models adjusted for demographics (i.e., age, sex), eGFR, LDL-C, average number of LDL-C measurements in the year prior, all comorbidities (history of previous MI, previous revascularization, diabetes mellitus, hypertension, heart failure, pad, valve disorder, stroke, TIA, atrial fibrillation, other arrhythmias, chronic respiratory disease, other lung diseases, venous thromboembolism, liver disease, cancer, fracture in previous year) medications (beta-blockers, calcium channel blockers, diuretics, renin-angiotensin system inhibitors, digoxin, nitrates, antiplatelet, anticoagulants, beta-2 agonist, anticholinergic inhalants, glucocorticoids, inhalants, oral glucocorticoids, NSAIDs, opioids) healthcare utilization (cardiovascular hospitalizations in the previous year, non-cardiovascular hospitalizations during the last year, outpatient contacts for cardiovascular causes during the last year, outpatient contacts for non-cardiovascular reasons in previous year, no. of unique dispensed drugs during the last year), calendar year and education level.

**Figure S1**. **Graphical depiction of longitudinal study design.**

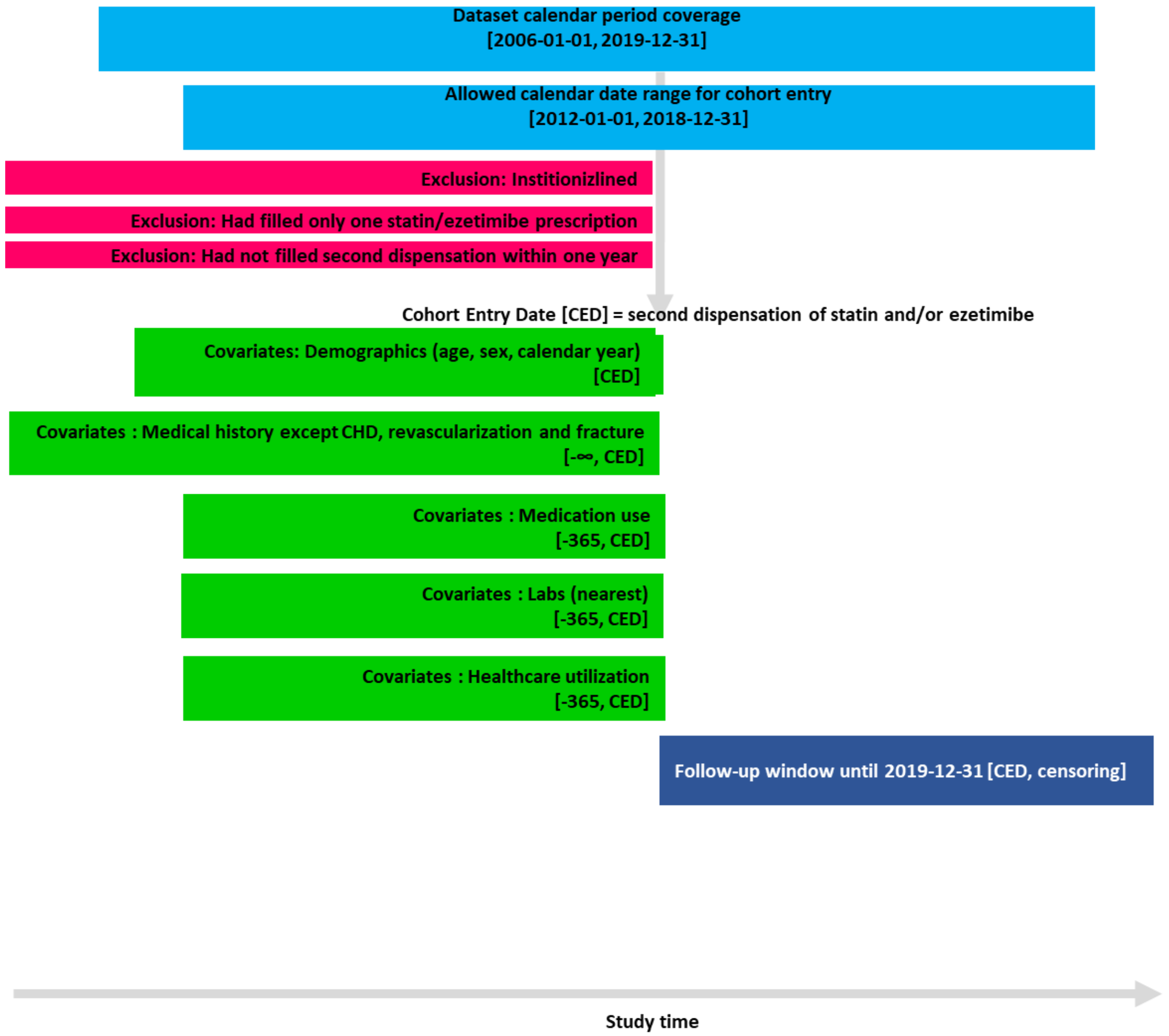

**Figure S2. Schematic representation of how the same individual may switch LLT exposures from one over follow-up time. LLT exposures with PDC ≥ 0.80 are adherent, 0 < PDC < 0.8 are poorly adherent and gaps of ≥ 1 year are discontinuation.**

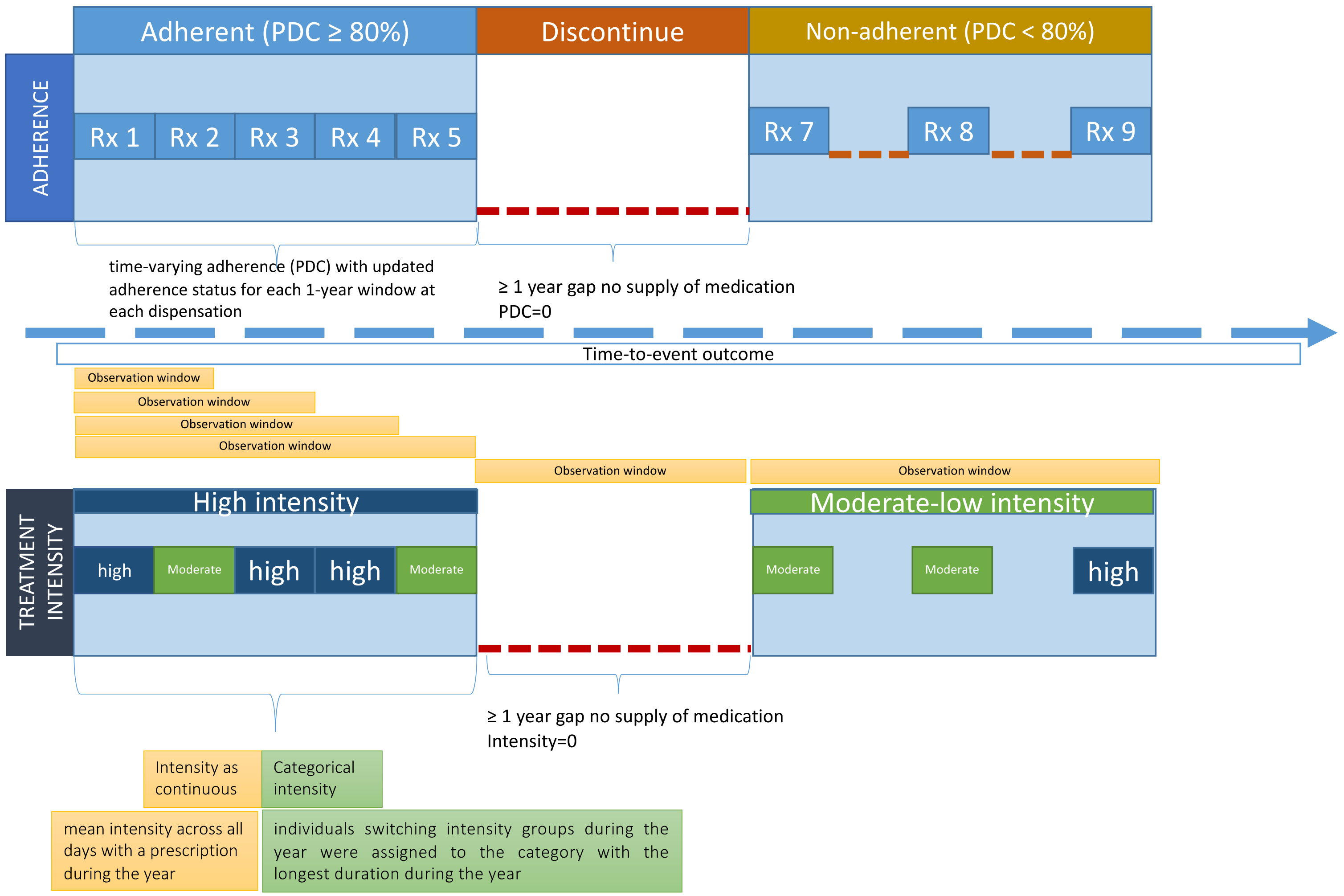

**Figure S3. Median and distribution of number of dispensations per patient during observation, by treatment intensity.**

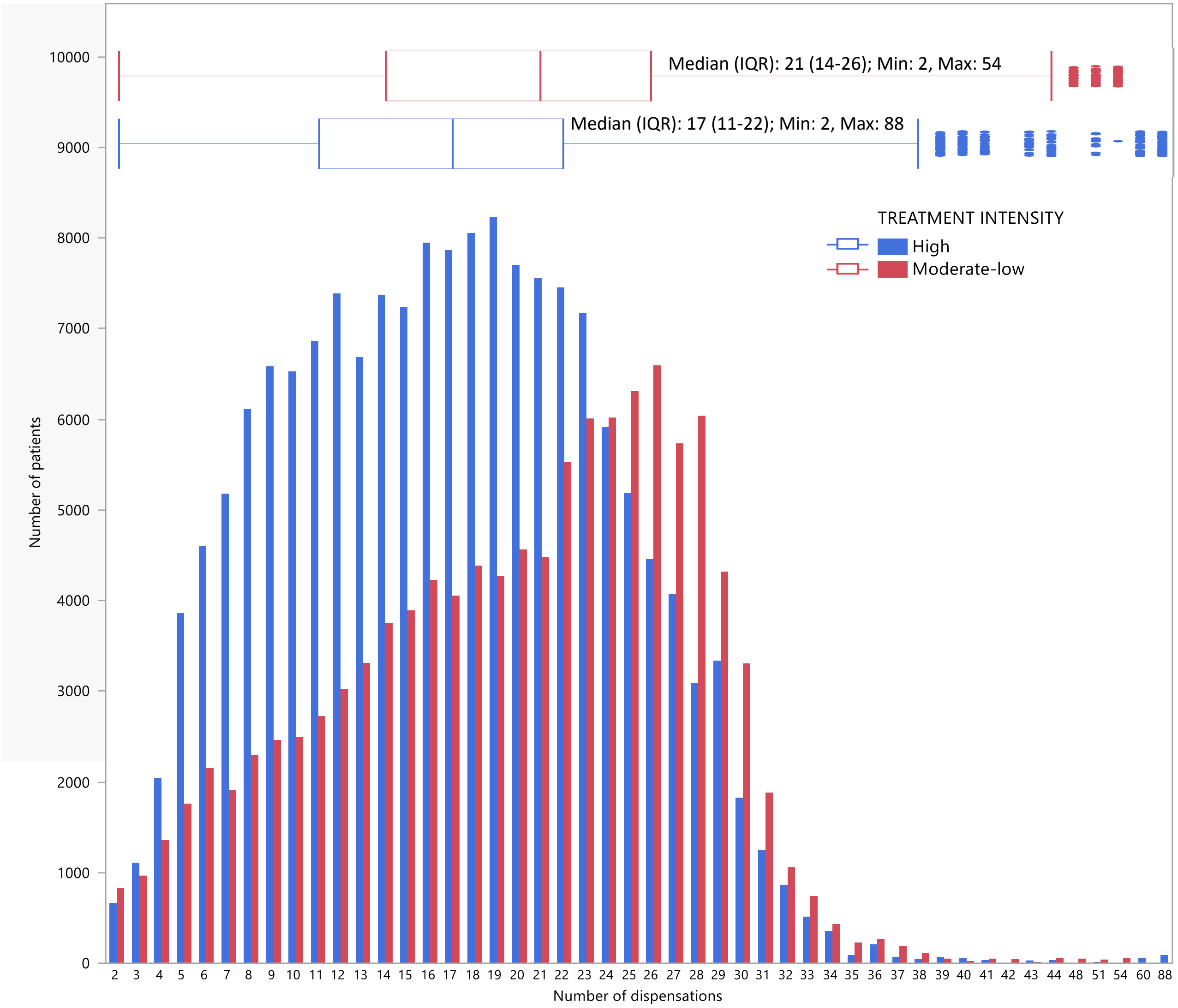

**Figure S4. Sensitivity analysis:** Graphical representation of the associations between continuous measures of statin (a) adherence, (b) intensity, (c) adherence and intensity-adjusted adherence, and the risk of MACE. Output from a sensitivity analysis which censored patients at treatment discontinuation.

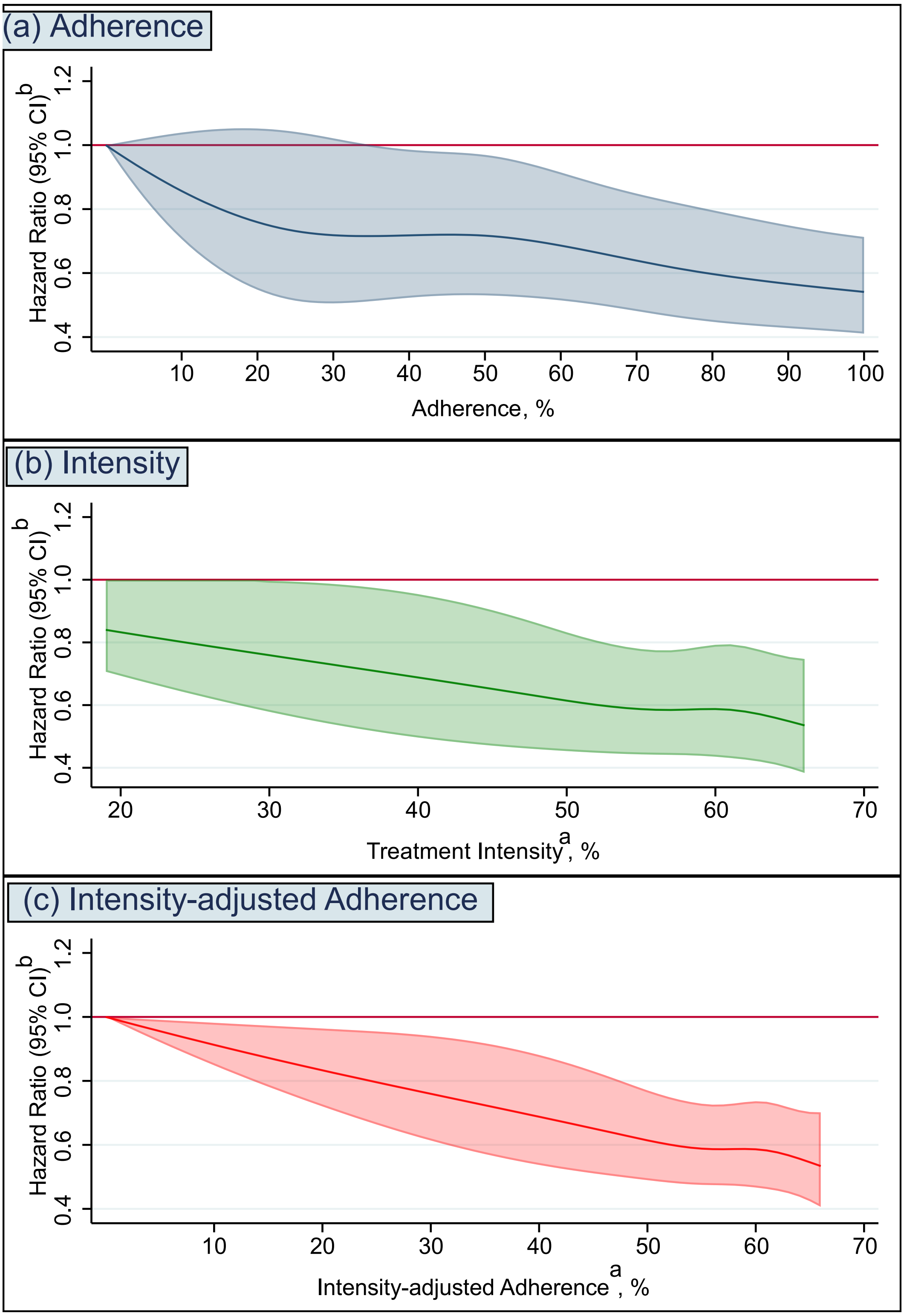


[a] The exposure statin intensity ranges from 0 to 66%, given that the highest doses of the most potent statins are estimated to lower LDL-C by 66% in clinical trials (See Supple table 1). The exposure of adherence

**Figure S5. Sensitivity analysis:** The effect of adherence (Y-axis, in %) on the predicted probability of LDL-C goal attainment across levels of intensity (X-axis, in %) and *vice versa*. Output from a sensitivity analysis which censored patients at treatment discontinuation.

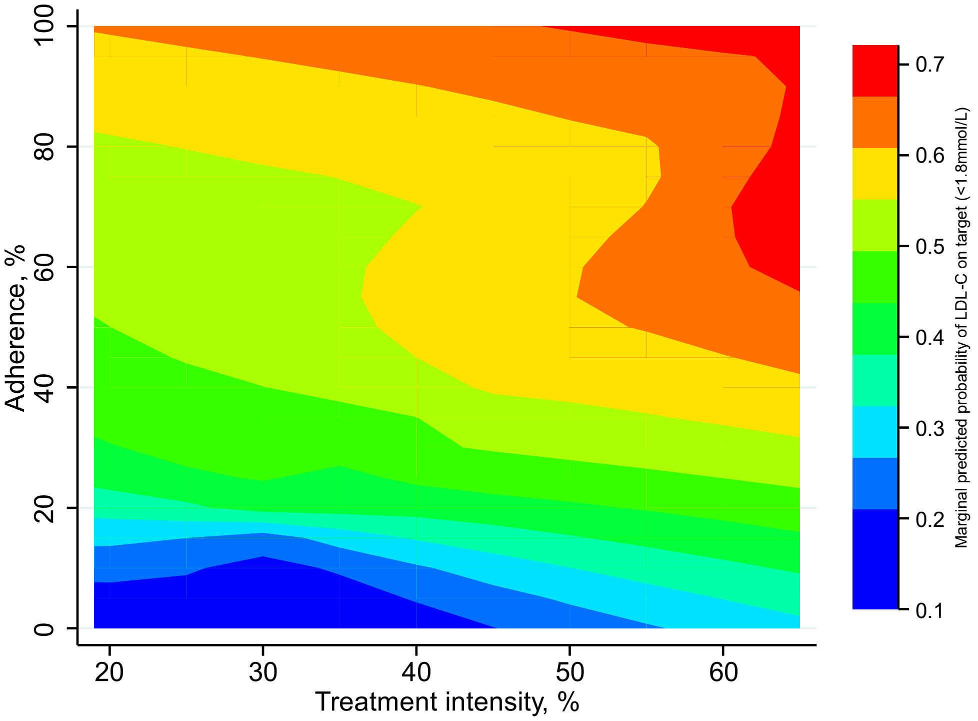


The exposure treatment intensity ranges from 19 to 66%, given that the highest doses of the most potent statins are estimated to lower LDL-C by 66% in clinical trials (See Supple table 1). The exposure of treatment adherence ranges from 0 to 100%.

Result of repeated-measures logistic regression with an interaction between two continuous covariates. The model includes subjects as random effects and fixed effect for patient demographics (i.e., age, sex), eGFR, average number of LDL-C measurements in the year prior, all comorbidities (history of previous MI, previous revascularization, diabetes mellitus, hypertension, heart failure, pad, valve disorder, stroke, TIA, atrial fibrillation, other arrhythmias, chronic respiratory disease, other lung diseases, venous thromboembolism, liver disease, cancer, fracture in previous year) medications (beta-blockers, calcium channel blockers, diuretics, renin-angiotensin system inhibitors, digoxin, nitrates, antiplatelet, anticoagulants, beta-2 agonist, anticholinergic inhalants, glucocorticoids, inhalants, oral glucocorticoids, NSAIDs, opioids) healthcare utilization (cardiovascular hospitalizations in the previous year, non-cardiovascular hospitalizations during the last year, outpatient contacts for cardiovascular causes during the last year, outpatient contacts for non-cardiovascular reasons in previous year, no. of unique dispensed drugs during the last year), calendar year and education level.
